# Food allergy and anaphylaxis – 2060. Oral Food challenges still the most reliable test for a diagnosis of food allergy

**DOI:** 10.1186/1939-4551-6-S1-P143

**Published:** 2013-04-23

**Authors:** Meera Thalayasingam, Michelle Meiling Tan, Cesar Brence Labastida, Lynette Shek Pei-Shi

**Affiliations:** 1 Department of Paediatrics, National University Hospital, Singapore

## Background

Food allergy in children is increasing in prevalence and severity. To parents this may translate to deliberate food restriction especially to either highly allergenic foods or to unknown foods (food neophobia). These claims need to be evaluated by a good clinical history and if warranted, skin prick testing and food specific IgE assays. However neither of these is diagnostic of food allergy. Therefore an oral food challenge (OFC) is indispensible in facilitating a diagnosis of true food allergy. The aim of this study is to examine the outcome of OFC based on the reason of avoidance.

## Methods

A retrospective chart review of all suspected paediatric IgE-mediated food allergym patients that underwent OFC administeredat the Allergy Unit National University Hospital, Singapore during a 2-year period.

## Results

A total of 197 challenges were performed in 58 patients.The median agewas 6 years and 58% were male. Atopic co-morbidity eczema was seen in 39.7%, asthma in 24.1% and rhinitis in 20.7% of patients. Forty-four percent of challenges were to foods that were never eaten, 26% of challenges were to foods due to a previous SPT and /or immunoassay results, 16% were to foods thought to have worsened their eczema and only 14% to foods thought to have caused a previous reaction. Previous reactions were reported as cutaneous (9.6%), oral (3%) and perceived anaphylactic reactions in 3%. Forty-three patients underwent multiple challenges. Of the 197 challenges, the majority was to tree-nuts in 54%, peanuts in 10% and shellfish in 8.5%. Of the 10 (5%) positive challenges, reactions were mostly cutaneous (urticaria and angioedema). No episodes of anaphylaxis were reported post challenge and no epinephrine was dispensed. Challenge positive subjects had either positive SPT (wheal > 3mm) or raised serum IgE levels to the specific food that they reacted to during the challenges.

## Conclusions

The use of restrictive diets and over-reliance on allergy tests in the absence of a history suggestive of clinical food allergy is of concern. Ninety-five percent of food challenges were negative and consequently these foods were introduced into the diet of most of our subjects.

